# Recurrent hyperammonaemia in a patient with carbonic anhydrase VA deficiency

**DOI:** 10.1002/jmd2.12322

**Published:** 2022-08-10

**Authors:** Christopher Stockdale, Ann Bowron, Marie Appleton, Ruth Richardson, Mark Anderson

**Affiliations:** ^1^ Department of Blood Sciences Newcastle upon Tyne Hospitals NHS Foundation Trust Newcastle upon Tyne UK; ^2^ Northern Genetics Service Newcastle upon Tyne Hospitals NHS Foundation Trust Newcastle upon Tyne UK; ^3^ Great North Children's Hospital Newcastle upon Tyne Hospitals NHS Foundation Trust Newcastle upon Tyne UK

**Keywords:** ammonia, CA‐VA, hyperammonaemia, urea cycle

## Abstract

Carbonic anhydrase VA deficiency is a recently described inherited cause of paediatric hyperammonaemia. Most published cases describe patients with only one episode of hyperammonaemia whilst others report patients who had up to three metabolic crises with the first invariably being the most severe. We describe a patient with carbonic anhydrase VA deficiency who experienced 7 hyperammonemic episodes over a 3‐year period, up to age 5 years 9 months. These episodes did not clearly decrease in severity over time. This report expands the clinical phenotype and the age window for metabolic crises associated with this condition.

**SYNOPSIS**

Carbonic anhydrase VA (CA‐VA) deficiency can feature multiple episodes of severe hyperammonaemia up to the sixth year of life.

## 1 INTRODUCTION

Carbonic anhydrase (EC: 4.2.1.1) VA (CA‐VA) provides bicarbonate for the mitochondrial enzymes carbamoyl phosphate synthetase 1 (CPS1), propionyl‐CoA carboxylase (PCC), 3‐methylcrotonyl‐CoA carboxylase (3MCC) and pyruvate carboxylase (PC).^1^ CA‐VA deficiency (OMIM 615751) has relatively recently become recognised as an inherited cause of episodic hyperammonaemia, to which inhibition of CPS1 is predicted to make the main contribution.^1^ Ketonuria and hyperlactatemia are consistent findings during hyperammonemic episodes.^1^, ^2^ The largest published case series described a maximum of three metabolic decompensations per patient with the majority experiencing a single event.^2^ In patients with multiple decompensations, the first event was invariably the most severe.^2^ Subsequent reports of CA‐VA deficiency have described either one or two episodes of hyperammonaemia in each affected patient.^3^, ^4^, ^5^, ^6^, ^7^ One of these articles did however feature a patient with recurrent hypoglycemia.^4^ We wish to highlight a case of CA‐VA deficiency featuring, to date, 7 episodes of hyperammonaemia (plasma ammonia >150 μmol/L) which did not clearly decrease in severity over time. The most recent episode occurred at age 5 years, 9 months which is over 18 months later in life than any other previously reported crisis in CA‐VA deficiency.

## 2 CASE PRESENTATION AND DISCUSSION

The patient was born at 33 weeks gestation in respiratory distress requiring continuous positive airway pressure (CPAP) and oxygen support on day 1 of life. Neonatology review at 3 months noted no concerns however there were multiple hospital attendances during the first 2 years of life due to bronchiolitis and gastroenteritis.

At 2 years 8 months he presented to the emergency department with reduced consciousness. He required intubation for respiratory depression and was transferred to the paediatric intensive care unit (PICU). Initial plasma ammonia was 311 μmol/L (reference range ≤50) which normalised rapidly following treatment with nitrogen‐scavenging medications and a single dose of *N*‐carbamyl‐l‐glutamate (NCG). Serum liver function tests were normal. Blood gas analysis showed metabolic acidosis with peak lactate 4.9 mmol/L (reference range ≤2.0). The patient spent 3 days on PICU and was discharged on glycerol phenylbutyrate (1.65 g, 3 times daily) with avoidance of excess dietary protein (target 0.9–1.0 g/kg/day) and an emergency regimen plan in case of illness. Sodium benzoate (500 mg, 3 times daily) was added later, following the hyperammonemic episode aged 3 years 10 months.

Metabolic investigations were requested during the first admission. A urine organic acid profile showed ketonuria and no clear diagnostic findings. Of relevance to the final diagnosis the 3MCC deficiency marker 3‐hydroxyisovaleric acid was mildly elevated but another 3MCC deficiency marker 3‐methylcrotonylglycine was not increased, nor were the PCC deficiency markers propionylglycine, 3‐hydroxypropionic acid and methylcitric acid. Bloodspot acylcarnitines also showed evidence of ketogenesis. Urine orotic acid (measured by stable isotope dilution gas chromatography–mass spectrometry) was not elevated. Urine and plasma amino acids also showed no diagnostic abnormalities. Plasma glutamine was 859 μmol/L (reference range 250–900), the peak plasma glutamine measured for this patient. Plasma alanine was initially within reference range and subsequently inconsistently elevated with peak concentration 1104 μmol/L (reference range 150–650), in a sample collected outside of a period of crisis. Analysis of a hyperammonaemia‐related disorders gene panel was performed, comprising 14 genes including those altered in the 6 known urea cycle disorders. No pathogenic variants were detected.

CA‐VA deficiency was not part of the hyperammonaemia gene panel, therefore analysis of the gene responsible (*CA5A*) was requested. This detected a homozygous deletion of *CA5A* exon 6, NM_001739.1:c.619‐3420_c.774 + 502del4078bp (p.Asp207_Gln258del), which had previously been reported, classified as pathogenic and predicted to be an inactivating variant.^1^, ^2^ Aged 4 years and 3 months the patient was thus diagnosed with CA‐VA deficiency.

In the 3 years following the initial hyperammonemic episode the patient had 6 more recorded episodes when plasma ammonia concentration peaked >150 μmol/L (Table 1), most recently at age 5 years 9 months, despite good compliance with diet and drug therapy, including the emergency regimen. Hyperlactatemia was detected during all but one of the admissions (Table 1), the highest being plasma lactate of 11.1 mmol/L (reference range 0.6–2.5). At least some of the decompensations were associated with infections. For example, a viral induced wheeze was noted during recovery from episode 1, and viral hand‐foot‐and‐mouth disease had been diagnosed prior to episode 3. He required PICU admission twice more and management on both occasions included hemofiltration; these episodes did not appear to be associated with late presentation, but a failure of conventional attempts to reverse hyperammonaemia with nitrogen scavenging drugs. NCG has been reported to be effective in CA‐VA deficiency^2^ and was administered in addition to intravenous nitrogen scavenging medications during episodes 1, 2, 6 and 7 (episodes 1–6 occurred before the diagnosis of CA‐VA deficiency had been made). Raised intracranial pressure and bilateral cerebral cortical cytotoxic oedema were detected during episode 2. Lactate and ammonia were within reference ranges between acute episodes. To date, hypoglycaemia has not been detected.

**TABLE 1:** Details of hyperammonemic episodes experienced by CA‐VA deficiency patient

Episode	Age	Peak ammonia (μmol/L)	Peak lactate (mmol/L)	Notes
1	2 years 8 months	311	4.9 (B)	PICU admission
2	2 years 10 months	261	11.1 (P)	PICU admission, hemofiltration, raised intracranial pressure, cerebral oedema
3	3 years 1 month	189	3.9 (B)	
4	3 years 4 months	153	5.0 (B)	
5	3 years 7 months	250	4.7 (B)	PICU admission, hemofiltration
6	3 years 10 months	181	3.3 (B)	
7	5 years 9 months	179	2.0 (B)^a^	

*Note*: Reference ranges: plasma ammonia ≤50 μmol/L; plasma lactate 0.6–2.5 mmol/L (P); blood lactate ≤2.0 mmol/L (B).

Abbreviation: PICU, paediatric intensive care unit.

a Lactate sample >4 h after peak ammonia.

The ketonuria and hyperlactatemia at the time of hyperammonaemia seen in this patient are in keeping with other case reports of CA‐VA deficiency, as are the results of metabolic biochemistry investigations.^1^, ^2^, ^3^, ^4^, ^5^, ^6^, ^7^ However the number, relative severity and age at occurrence of the hyperammonemic episodes are distinct from these previous reports. The majority of previously described CA‐VA deficiency patients had one metabolic crisis without recurrence.^2^, ^3^, ^4^, ^5^, ^6^, ^7^ The highest number of crises reported in any patient was 3 and any second and third crises were invariably less severe than the initial ones.^2^, ^4^ A suggested explanation for this was compensation for CA‐VA deficiency by maturation of the CA‐VB enzyme isoform.^2^ The previous maximum reported age for metabolic crisis in this condition was 4 years 1 month.^2^ By contrast the patient described here has had to date 7 separate episodes of hyperammonaemia which have not clearly decreased in severity over time. The patient was aged 5 years 9 months at the time of the most recent episode, extending the reported age range for metabolic crises in CA‐VA deficiency by over 1.5 years.

It should therefore not be assumed that the initial metabolic crisis in CA‐VA deficiency will be the only such event experienced by the patient, nor the most severe. Factors influencing the number and severity of metabolic crises in CA‐VA deficiency merit further study. Hyperammonaemia may occur at least as late as the sixth year of life in this condition; as a result, provision and update of an emergency regimen for acute illness management should be continued throughout childhood.

## AUTHOR CONTRIBUTIONS

Christopher Stockdale wrote manuscript, reporting of laboratory results. Ann Bowron manuscript revision, reporting of laboratory results. Marie Appleton manuscript revision, laboratory analyses. Ruth Richardson manuscript revision, patient management. Mark Anderson manuscript revision, patient management.

## FUNDING INFORMATION

No funding has been received for this study.

## CONFLICT OF INTEREST

Christopher Stockdale, Ann Bowron, Marie Appleton, Ruth Richardson and Mark Anderson declare that they have no conflict of interest.

## ETHICS STATEMENT

## INFORMED CONSENT

Signed parental consent for publication has been obtained.

## DETAILS OF ETHICS APPROVAL

Signed parental consent for publication has been obtained.

## PATIENT CONSENT STATEMENT

Signed parental consent for publication has been obtained.

## CARE AND USE OF LABORATORY ANIMALS

This article does not contain any studies with human or animal subjects performed by any of the authors.

## Data Availability

Not applicable as no datasets were generated or analysed during the current study.
